# An N400 identification method based on the combination of Soft-DTW and transformer

**DOI:** 10.3389/fncom.2023.1120566

**Published:** 2023-02-16

**Authors:** Yan Ma, Yiou Tang, Yang Zeng, Tao Ding, Yifu Liu

**Affiliations:** ^1^College of Computer and Information Science, Chongqing Normal University, Chongqing, China; ^2^Wisdom Education Research Institute, Chongqing Normal University, Chongqing, China

**Keywords:** event-related potential, dynamic time warping (DTW), self-attention, N400, transformer

## Abstract

As a time-domain EEG feature reflecting the semantic processing of the human brain, the N400 event-related potentials still lack a mature classification and recognition scheme. To address the problems of low signal-to-noise ratio and difficult feature extraction of N400 data, we propose a Soft-DTW-based single-subject short-distance event-related potential averaging method by using the advantages of differentiable and efficient Soft-DTW loss function, and perform partial Soft-DTW averaging based on DTW distance within a single-subject range, and propose a Transformer-based ERP recognition classification model, which captures contextual information by introducing location coding and a self-attentive mechanism, combined with a Softmax classifier to classify N400 data. The experimental results show that the highest recognition accuracy of 0.8992 is achieved on the ERP-CORE N400 public dataset, verifying the effectiveness of the model and the averaging method.

## Introduction

Cognitive linguistics, derived from cognitive science or cognitive psychology, is a subdiscipline of linguistics. The discipline focuses on describing and explaining the systemic nature, structure, and function of language (Turakhonova, 2022). Electroencephalography (EEG) is the sum of postsynaptic potentials of neurons in the human cerebral cortex, which is acquired through the brain-computer interface (BCI) at the surface of the scalp (Nicolas-Alonso and Gomez-Gil, 2012). In recent years, there has been a gradual increase in the number of language cognitive indicators built around EEG features, thanks to the high temporal resolution of EEG signals, the richness of the implicit information, and the non-invasive acquisition process (Sun et al., 2020). Among them, the time-domain feature event-related potentials reflecting the impact of discrete events on the cognitive processing of the human brain play an important role (Zhang et al., 2019).

N400 is a classical endogenous component of event-related potentials that are associated with the cognitive processing of language in humans (Lau et al., 2008). N400 was first identified by Kutas and Hillyard (1980). The duo modified the Oddball paradigm for linguistic material by attempting to induce the p3b event-related potential through the semantic inconsistency of words at the end of English phrases but unexpectedly found another large Unrelated potential effect that manifested as a relatively Unrelated peak around 400 ms after the subject’s brain received the relevant stimulus, hence the name N400 (Kutas and Hillyard, 1980). The N400 was shown to reflect the processing of text semantics and text context in the human brain. Several dimensions such as signal amplitude, latency, and scalp distribution reflect the characteristics and distinctions of the human brain’s semantic perception of things (Liang et al., 2018).

Today, the N400 is considered a powerful tool for studying how human language is understood and processed. The linguistic media involved include, but are not limited to, text, speech, and non-textual language, sometimes expressed in the form of graphics, etc. The different stages of semantic comprehension by the human brain can be tracked by the N400 with millisecond temporal resolution (Petten, 1993). Therefore, fast and effective detection and classification of N400 event-related potentials have become a necessary and urgent need. Some recent work has used neural language models (NLMs) to investigate the relationship between N400 event-related potential amplitude, onset time, and cognitive processes in human language. Schwartz and Mitchell (2019) proposed that RNN can explain the magnitude of N400 event-related potential amplitudes evoked during subjects’ reading; Aurnhammer and Frank (2019) used GRU, LSTM, and other models to predict N400 amplitudes; Merkx and Frank (2020) suggested that T-LMs models have higher prediction accuracy for N400 signal amplitudes; the research direction of the above works mainly focused on exploring how human language comprehension systems work through prediction of N400 data amplitudes. Solutions for the detection and classification of N400 event-related potential data are relatively scarce. Most event-related potential detection and classification algorithms to date have been based on experiments with the P300 data set, and there is still a lack of more mature solutions for the task of N400 event-related potential detection.

To address this problem, this study first briefly summarizes the existing solutions for identifying and classifying other event-related potential components. Researchers in the field of brain-computer interfaces have tried to improve the recognition of event-related potentials from several perspectives, including feature extraction methods and models. Gui et al. (2015) evaluated two sequence similarity measures, Euclidean distance as well as DTW, and obtained optimal classification results on the Oz electrode channel, where Euclidean distance outperformed DTW distance. In the study of Al-rubaye et al. (2019) the DTW based on connectivity features was used for the classification of single-trial ERP cycles making a combination of support vector machine and K-NN classifier using the DTW metric and obtaining more accurate classification results. Davis (2016) used a DTW-based center of gravity averaging method for EEG sequences and proposes several further modifications to the initial sequence selection process to improve the effectiveness of the method in EEG analysis. Simbolon et al. (2015) used support vector machines to classify P300 event-related potentials to distinguish whether subjects were lying or not and obtained a classification accuracy of 70.83%; Abou-Abbas et al. (2021) used K-Nearest Neighbor, support vector machine, and decision tree to identify N290 and P400 event-related potentials, and finally obtained 78.09% recognition accuracy by decision tree. Traditional machine learning classification methods are highly dependent on manual feature extraction from raw data, which is a cumbersome workflow, so researchers are gradually introducing deep learning methods into EEG analysis. Chou et al. (2018) combined the Inception v1 network and EEGNet network to design an improved CNN-IE network and obtained a correct rate higher than 95% on the P300 event-related potential data obtained by superposition averaging. Yu et al. (2021) used a one-dimensional time-space convolutional kernel for feature extraction of the P300 standard dataset and combined it with a support vector machine classifier for classification to achieve the effective classification of few-trial visual event-related potentials. Maddula et al. (2017) proposed a model architecture based on recurrent convolutional neural networks to achieve efficient detection of P300 event-related potentials. Wang et al. (2022) introduced an attention mechanism into a long and short-term memory recurrent neural network to fully capture the global time-domain information of P300 sequences and achieved a classification accuracy of 91.9%. Duru and Duru (2018) used topographic maps of P300 and P400 for their respective trigger periods as recognition features and achieved a classification accuracy of 73% for the training set.

The N400 signal is a non-strictly phase-locked event-related potential, and multi-trial averaging can easily lead to time-domain feature falsification, resulting in difficulty in feature extraction and model training underfitting (Kutas and Federmeier, 2011). Different event-related potential types, datasets as well as different acquisition methods can lead to large differences in the multidimensional characteristics of event-related potentials. The existing methods are not generalizable. Therefore, this study hopes to design a more effective classification scheme for N400 event-related potential identification based on the existing research results of event-related potential classification and identification, and try to use the N400 event-related potential dataset provided by the ERP CORE database as an entry point. This study proposes a Soft-DTW-based event-related potential averaging scheme for feature extraction of N400 data and a simplified Transformer neural network model. The main contributions of this study are as follows.

1) The Soft-DTW differentiable loss function is introduced into the feature extraction process of N400 data, which takes into account the time series translation invariance while accelerating the backpropagation speed by dynamic programming and improving the efficiency of data feature extraction.
2) A Soft-DTW-based single-subject short-range event-related potential averaging method is proposed to partially Soft-DTW average the N400 similar data within a single-subject range to achieve the maximum retention of the location information and curve features of the N400 time series under the condition of multi-trial averaging.
3) Based on the data features of N400 event-related potentials, a TransFormer-based event-related potential classification model is proposed to fully capture the temporal characteristics and contextual relationships of N400 data by introducing location coding and self-attentiveness mechanisms, achieving the highest recognition accuracy of 89.92% on the ERP-CORE N400 public dataset.

## Materials and methods

### Soft-DTW-based averaging method for N400 event-related potentials

The N400 is a non-strictly phase-locked event-related potential with certain timing deviations in the time domain dimension, and the application of the traditional arithmetic superposition averaging method to it easily leads to the degradation of the signal-to-noise ratio of the time series, which is manifested by the disappearance of local features or the introduction of dense noise. In this study, a soft DTW time-series differentiable loss function was introduced, and a soft DTW-based superposition averaging method was designed based on this method to perform gravitational superposition averaging on the original N400 data and to extract event-related potential averaging sequences with smoother waveform features and more uniform start times from 35 electrode dimensions, respectively. The method was named the single-subject short-distance event-related potential superposition averaging method.

#### Dynamic time warping

The Dynamic Time Warping (DTW) algorithm is a numerical distance measure for time series. The traditional Euclidean distance algorithm relies on point-to-point numerical comparisons and cannot match time series of different lengths. The DTW algorithm allows one-to-many mapping between series points and combines the idea of dynamic programming to achieve an efficient comparison of time series similarity, and nowadays it has become one of the most commonly used metrics to quantify the similarity between series (Cuturi et al., 2007).

Assume that the multidimensional time series of variable length take values in the range of Ω ∈ ℝ^*p*^. Therefore, the time series can be represented as matrices with p number of rows and different number of columns. For two p-dimensional time series *x* = (*x*_1_,…,*x*_*n*_) ∈ ℝ^p*n^ and *y* = (*y*_1_,…,*y*_*m*_) ∈ ℝ^p*m^ with lengths n and m, respectively, define the cost matrix Δ(*x*,*y*):=[δ(*x_i_,y_j_*)]_*ij*_ ∈ ℝ^n*m^, where δ is the differentiable cost function δ:ℝ*^p^*×ℝ*^p^*→ℝ_+_. The Euclidean distance is chosen as δ in most cases. Let *r*_*i*,*j*_ be the cost sum of the cost matrix from the upper left corner to any point [i,j], and the dynamic programming equation of the DTW algorithm can be expressed as:


$$r_{i,j}^{D\,T\,W}=\delta_{i,j}+\min\left\{r_{i,j-1},r_{i-1,j},r_{i-1,j-1}\right\}.$$

The DTW algorithm effectively takes into account the translational invariance of the time series in the time domain dimension and is therefore often used in classifiers such as K-NN, SVM, etc. Petitjean et al. (2011) first proposed to output the entire time series as a fitted loss to achieve superposition averaging of the time series. However, at the computational level, the DTW algorithm is not trivial and unstable when applied to back-propagation loss functions, a factor that hinders the application of the DTW superposition averaging algorithm.

#### Soft-DTW loss function

To solve the problem of the non-minimizability of the DTW algorithm, Cuturi and Blondel (2017) used Soft minimum instead of DTW minimum and designed the minimizable Soft-DTW loss function. Under the Soft-DTW method, the sum of costs A can be expressed as:


ri,jS⁢o⁢f⁢t⁢D⁢T⁢W=δi,j+min{ri,j-1,ri-1,j,ri-1,j-1}γ.

min⁡a1γ,…,an={mini≤n⁡ai,γ=0,-γ⁢log⁢∑i=1ne-ai/γ,γ>0.

Where γ is the smoothing parameter. When γ = 0 the algorithm is equivalent to the conventional DTW. Define the set *A_n,m_*⊂{0,1}^*n*×*m*^, where each element A is an alignment matrix for the sequence x, y. For a particular alignment matrix *A* = [*a*_*i*,*j*_],*A* ∈ *A*_*n*,*m*_, only the point (i,j) on the path from (1,1) to (n,m) has its *a*_*i*,*j*_ = 1, and all other points have *a*_*i*,*j*_ value 0. The path follows three directions of extension downward, rightward, and diagonal right until it reaches the lower right corner of the matrix. The cost sum under this path matrix can be expressed as ⟨*A*,Δ(*x*,*y*)⟩. Thus, the soft-dtw loss function is defined as:


d⁢t⁢wγ⁢(x,y)=min{⟨A,Δ(x,y)⟩,A∈An,m}γ=-γ⁢log⁡(∑A∈An,me-⟨A⁢Δ⁢(x,y)/γ⟩).

#### Soft-DTW back propagation

The final goal of Soft-DTW is to obtain the numerical distance between time series x and y. If y is regarded as the target series, all that needs to be computed for back propagation is the gradient to the time series x. The gradient is expressed through the chain rule as:


$$\begin{array}{ccc} \nabla_{x}d\,t\,w_{\gamma }\,\left(x,y\right) & = & {\left(\frac{\partial \Delta \,\left(x,y\right)}{\partial x}\right)}^{T}\,\left(\frac{\partial d\,t\,w_{\gamma }\,\left(x,y\right)}{\partial \Delta \,\left(x,y\right)}\right)\\ & = & {\left(\frac{\partial \Delta \,\left(x,y\right)}{\partial x}\right)}^{T}\,\left(\frac{\partial r_{n,m}}{\partial \Delta \,\left(x,y\right)}\right). \end{array}$$

Applying the same chain rule to the second term yields:


$$\frac{\partial r_{n,m}}{\partial \delta_{i,j}}=\frac{\partial r_{n,m}}{\partial r_{i,j}}\,\frac{\partial r_{i,j}}{\partial \delta_{i,j}}=\frac{\partial r_{n,m}}{\partial r_{i,j}}\cdot 1=\frac{\partial r_{n,m}}{\partial r_{i,j}}.$$

Define the elements $e_{i,j}=\frac{\partial r_{n,m}}{\partial r_{i,j}}$ such that *E* = [*e*_*i*,*j*_],*E* ∈ ℝ^*n*×*m*^ is a matrix consisting of *e*_*i*,*j*_. The above equation is then re-expressed as:


$$\nabla_{x}d\,t\,w_{\gamma }\,\left(x,y\right)={\left(\frac{\partial \Delta \,\left(x,y\right)}{\partial x}\right)}^{T}\,E.$$

In order to optimize the time complexity of back propagation, dynamic programming is introduced to calculate the matrix *E*. for matrix terms $e_{i,j}=\frac{\partial r_{n,m}}{\partial r_{i,j}}$, *r*_*i*,*j*_ are arithmetically associated with three terms *e*_*i*,*j* + 1_, *e*_*i* + 1,*j* + 1_, *e*_*i* + 1,*j*_, respectively, and according to the chain rule we get:


$$\begin{array}{c} \frac{\partial r_{n,m}}{\partial r_{i,j}}=\frac{\partial r_{n,m}}{\partial r_{i+1,j}}\,\frac{\partial r_{i+1,j}}{\partial r_{i,j}}+\frac{\partial r_{n,m}}{\partial r_{i,j+1}}\,\frac{\partial r_{i,j+1}}{\partial r_{i,j}}+\frac{\partial r_{n,m}}{\partial r_{i+1,j+1}}\,\frac{\partial r_{i+1,j+1}}{\partial r_{i,j}}. \end{array}$$

Thus, for the element *e*_*i*,*j*_ in the matrix *E*, the reverse derivation is performed using the following formula:


$$\begin{array}{c} a=e^{\frac{1}{\gamma }\,\left(r_{i+1,j}-r_{i,j}-\delta_{i+1,j}\right)},\\ b=e^{\frac{1}{\gamma }\,\left(r_{i,j+1}-r_{i,j}-\delta_{i,j+1}\right)},\\ c=e^{\frac{1}{\gamma }\,\left(r_{i+1,j+1}-r_{i,j}-\delta_{i+1,j+1}\right)},\\ e_{i,j}=e_{i+1,j}\cdot a+e_{i,j+1}\cdot b+e_{i+1,j+1}\cdot c. \end{array}$$

#### Soft-DTW-based time series averaging

Cuturi and Blondel (2017) proposed that Soft-DTW can be effectively used in the Fréchet averaging algorithm. Given N time series *y*_1_,……,*y*_*n*_, represented as N matrices *M*_1_,……,*M*_*n*_ with P number of rows and different number of columns (corresponding to imperfect matching of time series lengths), define a single center of gravity time series X and a set of normalized weights λ_1_,……,λ_*n*_ ∈ ℝ_+_, such that $\sum_{i=1}^{N}\lambda_{i}=1$. Assuming that X possesses a fixed length n, the objective of time series averaging with Soft-DTW is to solve the following optimization problem:


$$\min_{x\in {\mathbb{R}}^{p\times n}}\sum_{i=1}^{N}\frac{\lambda_{i}}{m_{i}}\,d\,t\,w_{\gamma }\,\left(x,y_{i}\right).$$

### Data preprocessing

**FIGURE 1:**
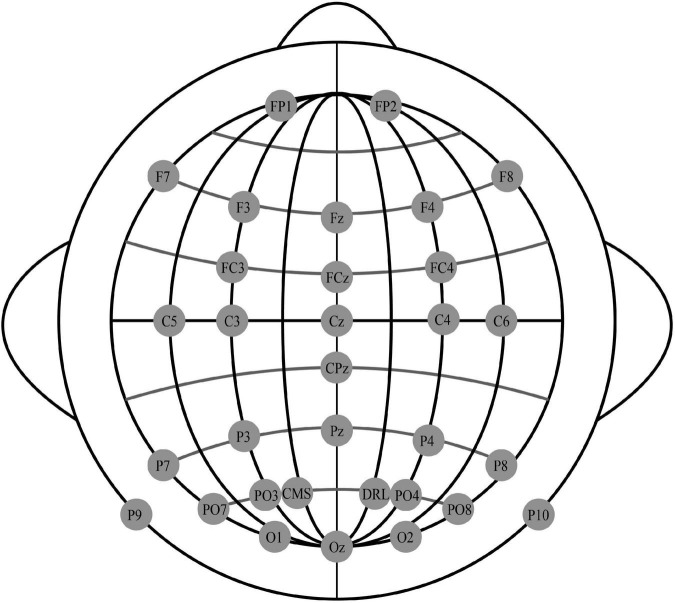
International 10–20 system.

The experimental dataset for this study was obtained from the N400 subset of the ERP CORE, an open EEG event-related potentials dataset provided by the University of California (Kappenman et al., 2021). The dataset was collected from 40 participants (25 females, 15 males; mean age 21.5 years, standard deviation 2.87 years, range 18-30 years; 38 right-handed) at the University of California, Davis, and all subjects were screened to ensure the normal or corrected-to-normal vision and normal color vision, with no history of neurological damage or disease. Thirty scalp electrodes were recorded using the Biosemi-ActiveTwo EEG signal recording system, with electrodes arranged according to the international 10-20 system, as shown in Figure 1.

**FIGURE 2:**
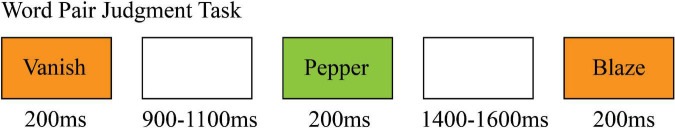
The example of the word pair judgment task information layout.

The experimental paradigm for the N400 sub-dataset uses a word pair judgment task adapted from that proposed by Holcomb and Kutas. In each trial, a red prime word was followed by a green target word. Participants answered whether the target word was semantically related or unrelated to the prime word, and labeled single events “Related” and “Unrelated” accordingly. In the process of data screening, subjects with artifacts higher than 25% were screened out. Subjects whose data will be excluded if their average accuracy on semantic judgments is less than 75%, or if their accuracy in any single experimental condition is less than 50%. An example of the word pair judgment task information layout is shown in Figure 2.

To eliminate the LCD delay, the stimulus event code was first shifted back to 26 ms on the time scale. the signal sampling rate was downsampled from 1,024 to 256 Hz to increase the data processing speed, and the data was re-referenced to the average of P9 and P10. To remove the DC offset present in the raw data, high-pass filtering was applied to the signal. In preparation for artifact correction, EEG data segments containing large myoelectric artifacts, extreme voltage shifts, and interruptions of more than 2 seconds were removed by visual judgment using the open-source tool ERPLAB. Subsequent independent component analysis of the data using the binICA method was used to remove artifacts and to remove components that were significantly correlated with horizontal eye movements (assessed by visual inspection of the scalp distribution of waveforms and components). The corrected bipolar HEOG channels (HEOG_left - HEOG_right) and VEOG channels (VEOG_lower - FP2) are calculated from the ICA correction data and the uncorrected bipolar HEOG and VEOG channels are retained for subsequent correction. Many studies have existed to fuse data from EEG with EEG as a way to improve the accuracy of interaction intent recognition. For example, Park et al. (2022) developed an online BCI appliance control system based on steady-state visual evoked potentials (SSVEP) and electrooculography (EOG); Karimi and Shamsollahi (2022) proposed a fiducial ensemble (Fpz-Cz, Pz-Oz, and EOG) based on latent structural influence models (LSIMs) that exhibited some degree of performance improvement. Therefore, we decided to use both EOG data for classification. The data were windowed based on the event list and baseline corrections were performed for each event point. Channels with excessive noise levels identified by visual inspection of the data were interpolated using EEGLAB’s spherical interpolation algorithm. The final EEG time-dependent potential data for 35 channels were retained [FP1, F3, F7, FC3, C3, C5, P3, P7, PO7, PO3, O1, Oz, Pz, CPz, FP2, Fz, F4, F8, FC4, FCz, Cz, C4, C6, P4, P8, PO8, PO4, O2, HEOG_left, HEOG_ right, VEOG_lower, (corr) HEOG, (corr) VEOG, (uncorr) HEOG, (uncorr) VEOG] totaled 4,497 entries, including 2,237 Related tags and 2,260 Unrelated tags. Each data is represented as a vector with a time series length of 256 and dimension of 35 (256 × 35). In this paper, experiments will be conducted based on the N400 sub-dataset of ERP CORE to verify the effectiveness of the method and model structure in identifying and classifying N400 data through the accuracy of the model in classifying differently labeled data.

### Single-subject short-range event-related potential averaging method

To extract features from the pre-processed N400 event-related potentials, this study proposes a feature extraction process called the “single-subject event-related potential averaging method” based on Soft-DTW (The following will be referred to as the SSE averaging method).

The data processing process was as follows.

1) Read the event-related potentials of 40 subjects separately, and classify the data into *P*_1_,……,*P*_40_ and *U*_1_,……,*U*_40_ according to “Related” and “Unrelated” labels within a single subject.
2) Taking *P*_1_ as an example, within this data grouping the DTW distances are calculated for every two units of data and saved as an array, named the distance matrix for this data set. The distance between two independent N400 data is calculated as follows: for two independent event-related potentials (256 × 35), each column is treated as a time series of length 256, and the standard DTW distance is calculated in EEG/EOG channels column by column and summed up, and saved in an equilateral array with side lengths of the divided data set as subscripts (the DTW distances of data 1 and data 3 are saved in the array subscripts [0][2] and [2][0] positions).
3) All data are traversed sequentially, and for each independent event-related potential signal *d*_1_, the N data with the smallest DTW distance within that data grouping (including *d*_1_ itself) is found by the distance matrix, and the N data are named as a similar sample group of *d*_1_.
4) Soft-DTW superimposed averaging is performed column by column for all data within similar sample groups in electrode channels, and the averaged results are output as a new sequence.

**FIGURE 3:**
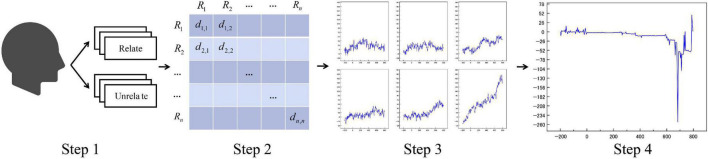
Simple schematic diagram of SSE averaging method.

The data processing flow is shown in Figure 3:

In Step 1, we restrict the data samples for each Soft-DTW superimposed averaging to be from the same subject and the same label category to retain enough individual subject characteristics and reduce the loss of useful temporal information. For example, subject 1 has 130 independent data, of which 70 are “Relate” labels and 60 are “Unrelate” labels. “Relate” data, if the number of similar sample groups N is set to 25, then within the 70 “Relate” labeled data, 24 of the closest data will be found to form a similar sample group with a total of 25 data based on the DTW distance, and the data within the similar sample group will be Soft -DTW averaging. In this way, similar data are averaged against each other to produce a new time series. As all data are averaged against their nearest data, the new average data are still somewhat data independent, avoiding excessive homogenization. So the feature processing process is named “single-subject short-range event-related potential superimposed averaging method “. The number N of similar sample groups for each independent sample should not be too large to mitigate subsequent model training overfitting. Each independent event-related potential signal will only be superimposed and averaged within the same scalp electrode, without any interference between data channels of different electrodes.

**FIGURE 4:**
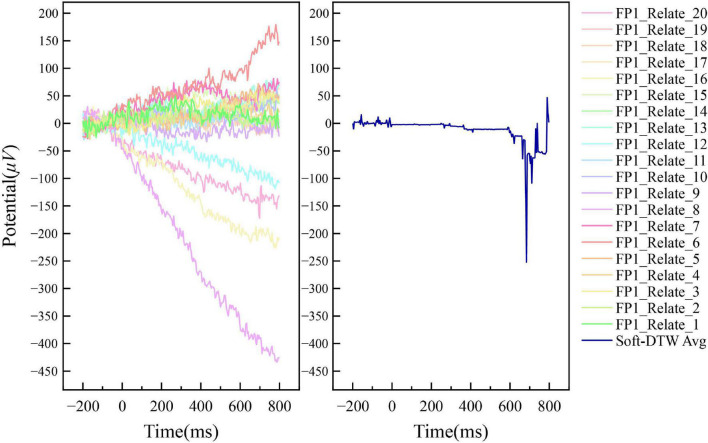
Example of the averaging results of N400 event-related potentials by Soft-DTW under FP1 channel (Related label).

**FIGURE 5:**
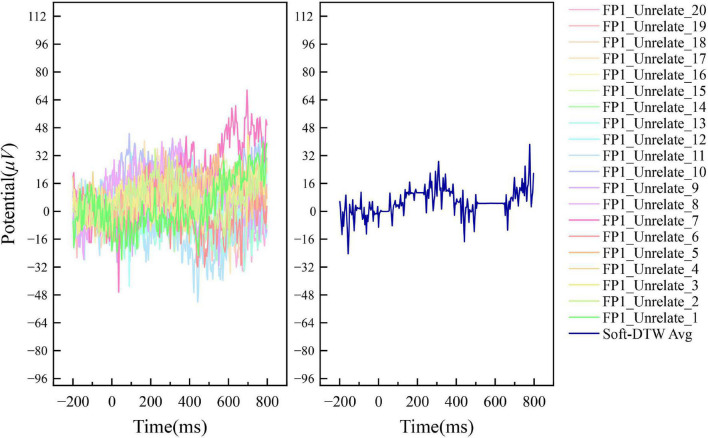
Example of the averaging results of N400 event-related potentials by Soft-DTW under FP1 channel (Unrelated label).

Figures 4, 5 show the results of the superimposed averaging of the random independent data on the FP1 electrode channel (the electrode position on the left side of the prefrontal lobe as defined in the international 10–20 system) for output subject 1 under the condition that the number of similar sample groups is 20. Where data FP1_Related/Unrelated_2 to FP1_Related/Unrelated_20 are the 19 data closest to the FP1_Related/Unrelated_1 data DTW of all signals acquired by the subject. The left subplot of the figure shows a general overview of the 20 data within this similar sample group, and the right subplot shows the Soft-DTW superimposed average results. In fact, the time series obtained using this averaging method are not consistent with the results of traditional event-related potential superposition averaging, and the standard event-related potential patterns are not usually observed in the time series waveforms extracted by the SSE averaging method. The main reason is that the method is not a global averaging but a limited Soft-DTW averaging over a similar sample set at the shortest DTW distance. This method is designed with the goal of serving the classification of N400 event-related potentials based on neural network models.

**FIGURE 6:**
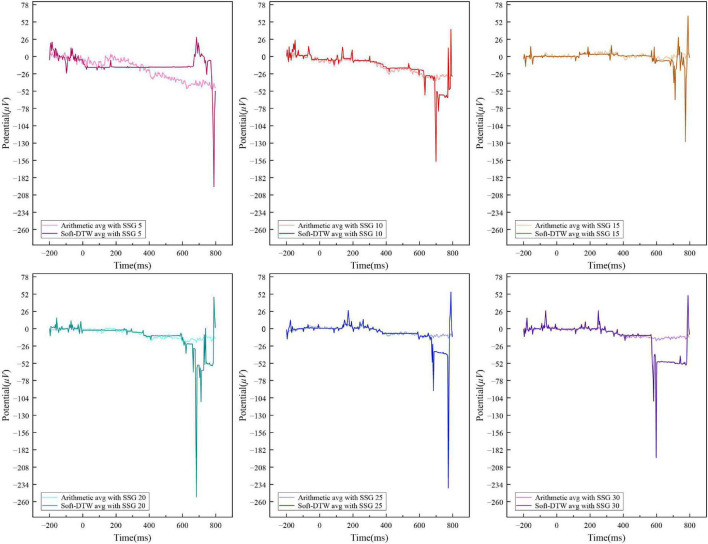
Comparison of the SSE-averaged and arithmetic-averaged results of Related label N400 data under different similar sample group numbers.

Figure 6 shows the sequence results obtained by SSE averaging and ordinary arithmetic averaging for the above random independent data with the number of similar sample groups of 5, 10, 15, 20, 25, and 30, respectively. The start time of the N400 data obtained based on the SSE averaging method advanced with the increase of the number of similar sample groups and the more similar samples were used for the superimposed averaging, the closer the averaging results were to the standard curve pattern of the N400 event-related potentials. The amplitude of the signal obtained by ordinary arithmetic averaging tends to flatten out with the increase in the number of similar sample groups, and the amplitude range is the same as that of the N400 signal data obtained by Unrelated labeling, which is difficult to distinguish effectively. The SSE averaging method can better capture the start-up utility of the N400 event-related potentials after the 400ms period, and the time series output extracted by this method is more suitable as a valid feature for the N400 event-related potential identification task.

### Transformer neural network

As an emerging deep learning model, Transformer has been widely used and achieved outstanding results in various fields such as natural language processing, speech processing, and computer vision. The Transformer was initially applied as a sequence-to-sequence model for machine translation tasks, and later many studies have shown that pre-trained models based on the Transformer can perform better on various tasks, and today Transformer has become one of the most commonly used model architectures in the field of natural language processing (Lin et al., 2022).

The traditional Transformer model consists of two main parts, the encoder and the decoder, each of which is a module with the same structure. The encoder consists of a self-attentive module and a feedforward neural network. To reduce the information loss in the deep network and to solve the problem of gradient disappearance and gradient explosion, residual links are introduced within the encoder. In contrast to the encoder, the decoder introduces an additional self-attentive module between the self-attentive block and the feedforward neural network, fuses the output of its self-attentive module with the output of the encoder and calculates the attention score, which is subsequently fed into the feedforward neural network. Finally, the decoder-processed features are fed into the fully connected layer to achieve the output of the sequence. The reason why Transformer outperforms RNN in predicting N400 amplitudes has been discussed by Michaelov et al. (2021) who concluded that the prediction results of N400 event-related potentials are influenced by the context, which has given some inspiration to this study. This study attempts to use a similar Transformer structure to capture the hidden contextual information inside the processed N400 event-related potential data and hypothesizes that this model can achieve more efficient and accurate identification of N400 data.

In this study, a simple Transformer neural network is designed for the N400 event-related potential features obtained by SSE averaging method, which is structurally streamlined and optimized for the N400 data features to achieve the effective classification of N400 signals within a limited batch.

#### Improved network architecture

**FIGURE 7:**
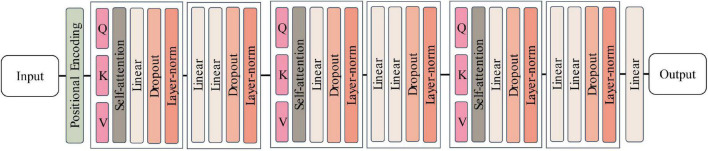
Erp-Transformer network structure.

In this study, a relatively simplified network architecture is proposed based on Transformer according to the data characteristics of N400 event-related potentials, and the network structure is shown in Figure 7. For ease of differentiation, the model structure is named Erp-Transformer in the following.

The network consists of three main modules, namely the position encoder module, the encoder group module, and the fully connected module. The internal structure of each module is described as follows.

Positional encoding module: N400 event-related potentials belong to typical time series features, and the purely self-attentive mechanism cannot fail to capture the position information contained in N400, including absolute position information, relative position information, and distance between different data points, so the position encoding module is introduced. The position encoding is calculated as follows: for a p-dimensional time series *x* = (*x*_1_,…,*x*_*m*_) ∈ ℝ^p*n^ of length n, define t as a 35-dimensional vector of event-related potentials at a certain moment, i as the ordinal number of the vector in the time series, and $\vec{d_{t}}\in {\mathbb{R}}^{p}$ denotes the position encoding vector corresponding to position t. Define the generating function *f* of the position vector $\vec{d_{t}}$:


$${\vec{d_{t}}}^{\left(i\right)}=f\,{\left(t\right)}^{i}:=\left\{ \begin{array}{c} \sin\left(\omega_{k}\cdot t\right),\text{if}\,i=2\,k,\\ \cos\left(\omega_{k}\cdot t\right),\text{if}\,i=2\,k+1. \end{array}\right.$$

The input sequence x enters the position encoding module to first obtain the position encoding sequence D by the above function calculation, and then the matrix addition operation is performed between x and D. The operation result is input to the Dropout layer, and the neuron information is randomly removed with a probability of P = 0.5 to mitigate overfitting. Finally, the operation result is input into the encoder group module.

**FIGURE 8:**
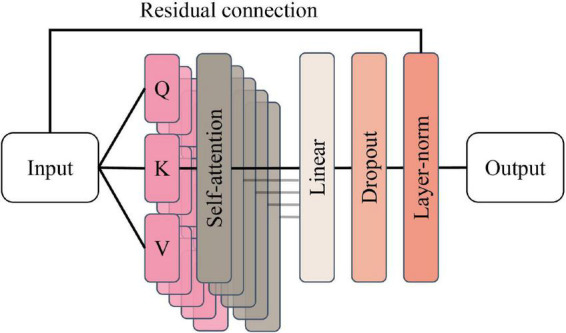
Multi-headed attention layer structure.

Encoder group module: The encoder group module contains three encoders with the same structure, and a single encoder contains a multi-headed attention layer and a position-encoding feedforward neural network inside, and the structure of the multi-headed attention layer is shown in Figure 8.

The multi-headed attention layer contains Num = 5 attention heads, denoted as *a* ∈ {1,…,*Num*}. Each attention head dimension is Dim = 35. Define the fully connected layers *L*_1_, *L*_2_, *L*_3_ with both input and output dimensions of 35, and convert the 35-dimensional vectors of a single moment into Q, K, and V vectors, respectively, calculated as follows.


$$Q_{\left(t\right)}^{\left(a\right)}=W_{Q}^{\left(a\right)}\,L_{1}\,\left(t\right)\in {\mathbb{R}}^{D\,i\,m}.$$

$$K_{\left(t\right)}^{\left(a\right)}=W_{K}^{\left(a\right)}\,L_{2}\,\left(t\right)\in {\mathbb{R}}^{D\,i\,m}.$$

$$V_{\left(t\right)}^{\left(a\right)}=W_{V}^{\left(a\right)}\,L_{3}\,\left(t\right)\in {\mathbb{R}}^{D\,i\,m}.$$

The Q, K, and V vectors will be used simultaneously to calculate the attention scores. Defining the scaling factor *D_k_* = 5^–0.5,^ the attention score is calculated as follows.


$$A\,t\,t\,e\,n\,t\,i\,o\,n\,\left(Q,K,V\right)=\text{softmax}\,\left(\frac{Q\,K^{T}}{\sqrt{D_{K}}}\right)\,V.$$

The result of the operation is first input to the fully connected layer for regularization, and the Dropout layer with P = 0.5 randomly removes the neuron information; then the residual connection operation is introduced to add the output of the Dropout layer directly with the input of the multi-headed attention layer in a matrix, and finally, the output data is LayerNorm normalized and input to the next layer of the network.

**FIGURE 9:**
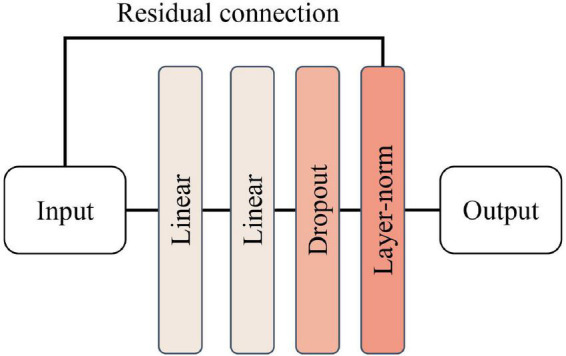
Structure of position-encoded feedforward neural network.

The structure of the position-encoded feedforward neural network is relatively simple and is shown in Figure 9, consisting of two fully connected layers, one Dropout layer (P = 0.5) and LayerNorm layer, respectively, with the Relu activation function introduced in the middle of the two fully connected layers to fine-tune the parameters. The purpose of this layer design is to learn the position information features introduced in the input features in combination with the position encoding module.

Fully connected module: The final module of the model consists of a single fully connected layer with an input dimension of 256 × 35 = 8,960 and an output dimension of N400 induced event category number 2. This module no longer directly uses the Relu activation function for parameter fine-tuning and directly outputs two-dimensional classification results.

Compared to the traditional Transformer structure used for machine translation tasks, the Erp-Transformer proposed in this paper has been somewhat simplified in structure. Compared to the original Transformer which uses six encoders and decoders and eight parallel attention heads (Vaswani et al., 2017), Erp-Transformer retains only three structurally identical encoders and the number of parallel attention heads in the multi-headed attention module is reduced to five. In the subsequent experimental validation section we learn that for the N400 features extracted by the SSE averaging method, the model proposed in this paper can also achieve good recognition and classification results with a smaller number of training parameters, smaller computation, and faster training speed.

### Experimental design

#### Experimental environment

The experiments were conducted on a Windows 10 Education (64-bit) computer operating system with an AMD Ryzen 5 1,600 Six-Core Processor and an NVIDIA GeForce RTX 2060 GPU, using Python 3.8.0 as the programming language. The experimental model was built based on the Pytorch machine learning framework.

#### Model evaluation

The following quantifiers were used in this study: correctly identified Relate N400 signal (True Relate, TR), correctly identified Unrelate N400 signal (True Unrelate, TU), incorrectly identified Relate N400 signal (False Relate, FR), incorrectly identified Unrelate N400 signal (False Unrelate, FU). Further define the N400 event-related potential recognition Accuracy, Precision, and Recall as:


$$A\,c\,c\,u\,r\,a\,c\,y=\frac{T\,R+T\,U}{T\,R+T\,U+F\,R+F\,U}$$

$$P\,r\,e\,c\,i\,s\,i\,o\,n=\frac{T\,R}{T\,R+F\,R}$$

$$R\,e\,c\,a\,l\,l=\frac{T\,R}{T\,R+F\,U}$$

#### Experimental details

Prior to model training, DTW distances were first computed separately for all data within the same label range for a single subject and stored as a matrix. The distance matrix was then used as the basis for feature extraction of all data using the Soft-DTW-based N400 event-related potential averaging method proposed above. After each individual event-related potential data matrix has passed through the entire SSE averaging process, a total of 4,497 corresponding superimposed averaging sequence matrices are output, with the total amount of data and data specifications remaining unchanged from the original data and still stored independently on a subject by subject basis.

This study will use 5-fold cross-validation to evaluate the performance of the model on this training set as well as its generalization ability. We divide the dataset into 5 copies on a subject-by-subject basis, each containing data from 8 subjects. One of these was selected at each time as the test set, and the remaining four were used as the training set for model training. Under a particular experimental condition, a total of five training sessions were conducted to obtain 5 × 100 epochs = 500 experimental results, with each experimental data containing four components: TR, TU, FR, and FU. Accuracy, Precision, and Recall were calculated separately for each training result, and the results obtained from the five training sessions were averaged within the same epoch to obtain the mean values of the three evaluation metrics under each epoch, and subsequently, the epoch data with the highest value was taken as the optimal average experimental result.

To make the model training effect optimal, the main parameters associated with the Erp-Transformer neural network were screened in this study, and the following optimal network parameters were finally determined: a random deactivation ratio of 0.5 for the Dropout layer, a learning rate of 5e-4 for the Relu activation function, 3 encoder layers for the encoder group, and 5 self-attentive heads for each independent encoder. The number of training data set batches is 256; the model training efficiency and accuracy perform best when the number of similar sample groups in the SSE average process is 25.

## Results and discussion

### Analysis and discussion of experimental results

**FIGURE 10:**
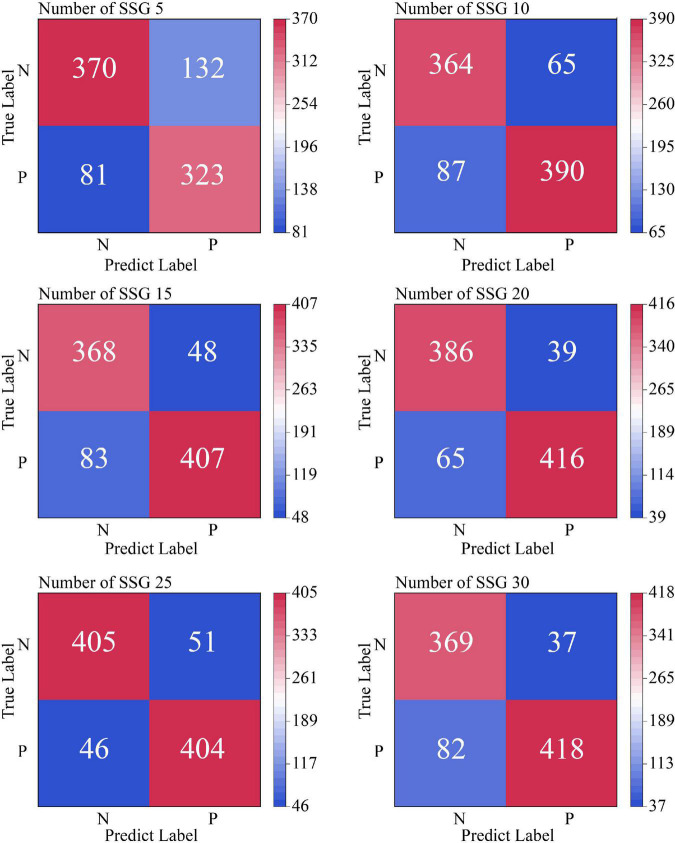
Confusion matrix for identifying classification results from test set N400 data under non-cross-validation conditions. In this figure, SSG is an abbreviation for similar sample groups.

**FIGURE 11:**
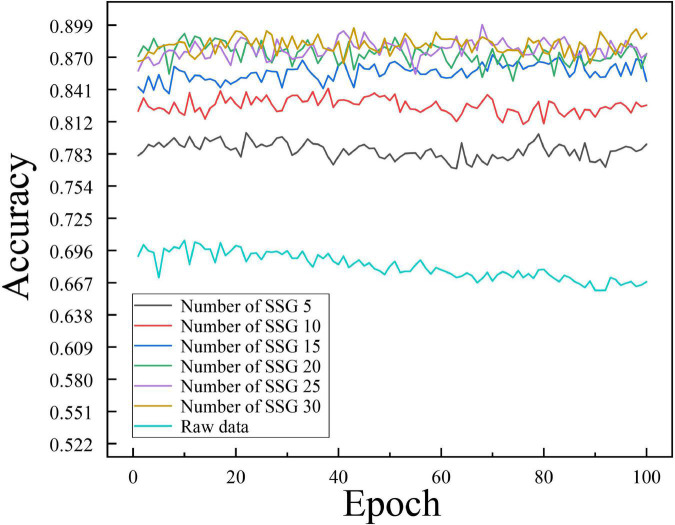
Variation of test set recognition accuracy (Accuracy) with training batches under different number of similar sample groups.

To investigate the influence of the number of similar sample groups of independent samples in the averaging process on the training effect of the model, we conducted comparison experiments between the SSE averaging results and the original data on the Transformer model for the number of similar sample groups of 5, 10, 15, 20, 25, and 30, respectively. Figure 10 shows the non-cross-validated confusion matrix of recognition classification results for the test set N400 data under the condition of a different number of similar sample groups. Figure 11 shows the test set recognition accuracy (Accuracy) line graph with training batches under a different number of similar sample groups with 5-fold cross-validation condition.

According to the experimental results shown in Figure 10, the Soft-DTW-based SSE averaging method effectively improves the model’s recognition of N400 data, and there is a Related correlation between the average recognition accuracy and the number of similar sample groups. From the experimental results shown in Figure 11, it can be seen that the original N400 time-correlated potential data without averaging reached the highest average Accuracy of 0.705 at the 10th training, and continued to show a certain degree of overfitting in the late training period. When the number of similar sample groups exceeded 20, the model recognition accuracy no longer improved significantly. The model with similar sample group number 25 condition reached the highest average Accuracy of 0.8992 at the 68th training, which is the highest recognition accuracy of the model compared to other similar sample group parameter conditions.

**FIGURE 12:**
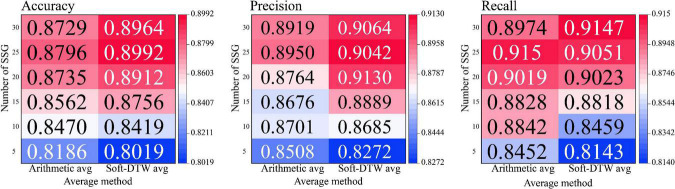
Hotspot of model recognition accuracy under different number of similar sample groups.

Since the act of superimposed averaging inherently leads to the proximity of data features within similar sample groups to each other, to demonstrate the effectiveness of the method used in this paper, we used applied the operational procedure of single-subject short-distance averaging to conventional arithmetic averaging and compared its obtained output combined with the recognition classification effect of the Transformer model with the Soft-DTW-based SSE averaging method, and the experimental results are shown in Figure 12.

After the experimental results described in the above table, it is known that the classification recognition results obtained by Soft-DTW applied to the single-subject short-distance averaging method are significantly higher than the arithmetic averaging method in terms of the Accuracy index. Under the condition that the number of similar sample groups is 25, the SSE averaging results obtained the highest recognition accuracy and precision, but when the number of sample groups is lower than 25, the arithmetic averaging method obtained better recall results. When the number of similar sample groups is small (below 10), the arithmetic averaging method can obtain higher recognition accuracy, however, when the similar sample groups contain more samples, the SSE averaging method shows better resistance to overfitting and higher recognition accuracy, precision, and upper recall. the high generalization of the SSE averaging method under 5-fold cross-validation indicates that it is more suitable for the N400 event-related potentials for high-precision recognition tasks.

In order to evaluate the recognition of N400 signals with features extracted by the SSE averaging method under various models, this study conducted controlled experiments using the Erp-Transformer neural network model against the mainstream event-related potential methods under the optimal condition of a similar sample group size of 25. We chose the CNN-IE model proposed by Chou et al., the Conv1d-based Erp feature extraction module proposed by Yu et al., the Conv2d+LSTM model used as a comparison in their work by Maddula et al. (2017) and the traditional Transformer network structure (Vaswani et al., 2017). In reproducing the above network structures, we uniformly used an input size of BatchSize × 256 × 35, retained the original number of channels, convolutional kernel size, and pooling layer parameters of the convolutional layer, and linked the above models to the exact same Softmax binary classification module (1,024 × 2) as Erp-Transformer to compare each model for N400 event-related potentials. The Accuracy, Precision, Recall, computation, number of parameters, and training time for each model with N400 data input were calculated for the arithmetic averaging method and the Soft-DTW averaging method, respectively.

According to the experimental results in Table 1, Conv1d and CNN-IE models performed better than Conv2d+LSTM models in N400 event-related potential recognition task, which was due to the fact that these two models had shown better performance in the same type of P300 event-related potential recognition task. However, their performance in identifying N400 event-related potentials is still inferior to that of Erp-Transformer and traditional Transformer. We can observe that the self-attention mechanism generally performs better on this data set. Regardless of the model, the short-distance event-related potential averaging method based on Soft-DTW showed significantly better recognition performance than the arithmetic averaging method.

**TABLE 1:** Comparison of the method of this study with other methods (when the number of similar sample groups is 25).

	Arithmetic avg			Soft-DTW avg		
Deep learning model	Accuracy	Precision	Recall	Accuracy	Precision	Recall
Conv1d	0.8480	0.8979	0.8804	0.8726	0.9122	0.8865
Conv2d+LSTM	0.6779	0.8000	0.6860	0.7837	0.9028	0.8180
CNN-IE	0.8221	0.9059	0.8653	0.8504	0.9166	0.9026
Transformer	0.9059	0.9063	0.9308	0.8966	0.9141	0.6860
Erp-Transformer	0.8943	0.9076	0.9188	0.8992	0.9042	0.9051

From the experimental results in Table 2, we know that the Conv1d model has the lowest hardware and time requirements for training, but the overall training effect is poor; the rest of the model methods containing 2D convolution have too many operations, a large number of parameters and relatively long training time. In contrast, the Erp-Transformer model has less computation, less number of parameters required, faster training efficiency, and relatively higher average recognition accuracy. Even though the recognition and classification results of the traditional Transformer model on the processed N400 dataset are comparable to the Erp-Transformer model proposed in this paper, it shows slightly better results under the arithmetic average condition. However, the traditional Transformer, because of its relatively complex network structure (6 encoders, 6 decoders, and 8 parallel self-attentive heads), increases the number of parameters and operations required for training substantially, and the average training time for 100 epochs is almost 7 times longer than that required by the Erp-Transformer. In contrast, the Erp-Transformer model is more cost-effective in achieving high recognition classification accuracy and is more suitable for recognizing N400 event-related potentials.

**TABLE 2:** Number of floating point operations per second (FLOPS), number of parameters, the time required for training per 100 epoch (in seconds) for model training under different methods.

	Conv1d	Conv2d+LSTM	CNN-IE	Erp-Transformer	Transformer
Flops	4.06 MMac	867.45 MMac	140.23 MMac	58.83 MMac	242.81 MMac
Params	2.59 M	366.74 k	127.58 M	329.6 k	1.11 M
Times	57.71 s	143.73 s	410.39 s	311.04 s	1741.92 s

## Conclusion

The identification and classification of N400 event-related potentials is important for cognitive linguistics as well as cognitive psychology research. Most of the existing event-related potential classification models are designed and tested based on the P300 dataset. Due to the large differences in multidimensional features of different event-related potential datasets, the models often have poor migration ability. Therefore, in this study, we propose a Soft-DTW-based single-subject short-range event-related potential averaging method for feature extraction of the original N400 data and a Transformer-based event-related potential classification model for the ERP CORE N400 event-related potential data set. The comparison test shows that the number of similar sample groups in the averaging method is closely related to the recognition accuracy of the model, and the Soft-DTW-based event-related potential averaging method is more beneficial to the recognition and classification of N400 data by the later model than the traditional arithmetic averaging method.

On the other hand, the Soft-DTW-based averaging method still has the disadvantages of low data processing efficiency and high time complexity when applied to event-related potentials, which makes the time cost of preliminary data processing high and reduces the overall recognition efficiency, so there is still much room for optimization of the overall data processing process of the method. At the same time, whether the method is compatible with other types of event-related potentials, and whether the method can be used as a data enhancement method to further improve the generalization of the model rather than just as a means of feature extraction, will be the focus of our next research.

## Data availability statement

The original contributions presented in this study are included in the article/supplementary material, further inquiries can be directed to the corresponding author.

## Ethics statement

Written informed consent was obtained from the individual(s) for the publication of any potentially identifiable images or data included in this article.

## Author contributions

YT was responsible for the design and implementation of the experiments and the overall writing of the manuscript. YM was responsible for the review and revision of the manuscript. YZ, TD, and YL were responsible for some of the programming and data visualization. All authors contributed to the article and approved the submitted version.
